# Type 2 diabetes and mortality in females versus males in England: the Salford diabetes cohort

**DOI:** 10.1097/XCE.0000000000000276

**Published:** 2023-01-04

**Authors:** Adrian H. Heald, Mike Stedman, Ian Laing, Martin Gibson, Martin Whyte

**Affiliations:** a The School of Medicine and Manchester Academic Health Sciences Centre, Manchester University, Manchester; b Department of Endocrinology and Diabetes, Salford Royal Hospital, Salford; c Res Consortium, Andover, Hampshire; University of Manchester, Manchester; d Department of Biochemistry, Royal Preston Hospital, Preston; e Clinical and Experimental Medicine, University of Surrey, Guildford, UK

Diabetes continues to be linked to a mortality rate that is greater than the background population. A previous study based on data from the National Health and Nutrition Examination Survey found that from 1971 to 2000, men with diabetes experienced a 43% relative reduction in mortality [1]. On the other hand, women with diabetes did not experience a similar decrease in mortality over this period, but instead experienced a doubling of the difference in mortality rate for women with diabetes compared with women without diabetes. The disparity in mortality rates by sex was driven by a wider gap in cardiovascular disease among women.

All-cause standardised mortality rate (SMR) is an effective measure for capturing the overall impact of long-term conditions on populations. Studies have shown that, overall, the SMR for people with diabetes in England is 1.5–1.7 [2,3] with significant differences in mortality rate by ethnic group [4]. There has been little work examining the factors that could be having an impact on this, nor on what may determine sex differences in outcome. Primary care data provides an invaluable resource to address these questions.

Patient data for the Salford region of England were extracted for patients with a diagnosis of type 2 diabetes (T2DM) in 2010 for the years up to 2020 including any deaths recorded [5]. The data extract was approved by the local research governance panel (application SIR022). Annual expected deaths were calculated from the annual Office of National Statistics mortality rate and life expectancy, by age and sex, relative to lower layer super output areas (LSOAs) [6], from which the Indices of Multiple Deprivation (2019) [7] were obtained.

Linear regression was used to establish the relation between Index of Multiple Deprivation (IMD) and local SMR over the study period. This formula was applied to adjust the expected national mortality rate by age and sex for each patient for their local LSOA IMD. Comparing the sum of the expected patient mortalities with the actual deaths gave an SMR with deprivation (SMRd) for any selected cohort.

We proceeded to examine the effect of sex differences on mortality rate over the 10 years period.

The study included 9558 people with 88 102 patient years. There were 2909 recorded deaths against 1851 expected, with SMR male (M) at 1.46 and for females (F) higher at 1.72 (+18%). SMR for diabetes varied by age group, being higher in younger individuals with diabetes relative to those without diabetes. In those with diabetes, the SMR was relatively higher in younger women than younger men. Specifically, for the 33% of deaths of T2DM patients at age less than 75 years, the SMR was 1.77 (M: 1.63 and F: 2.04). For the 37% of deaths at ages 75–84, the SMR was 1.4 (M: 1.27 and F: 1.58); and for the 30% of deaths at age 85 or more, the SMR was 1.10 (M: 1.05 and F: 1.15). Thus, the sex difference in SMR diminished with advancing age.

The percentage prescribed use of each drug as at least one of their medications was based on patient years use – either on their own or in combination: 85% had metformin with SMRd = 1.4 and 31% had sulfonylureas SMRd = 1.61. A total of 17% had a dipeptidyl peptidase-4–inhibitor SMRd = 1.57, whereas the 5% treated with sodium–glucose cotransporter 2 (SGLT2)–inhibitors had a relatively lower SMRd of 1.23. Drug use was similar between men and women except for SGLT2 inhibitors, where prescribing was lower in women (46%) versus men (54%). Patients on insulin plus oral treatment had much higher SMRd than oral alone, SMRd = 1.95.

In multiple logistic analysis the factors most strongly associated with death were the presence of diabetes foot complications (including foot ulceration) [odds ratio (OR), 6.3; 5.6–7.1], age at diagnosis of more than 65 years (OR, 2.6; 2.3–2.9), elevated urine albumin/creatinine ratio of more than 3 mg/mmol (OR, 1.7; 1.5–2.4), and estimated glomerular filtration rate less than 60 ml/min/1.73 m^2^ (OR, 2.3; 2.1–2.6). HbA1c, dyslipidemia, and hypertension were not found to be significantly predictive.

We have provided evidence that the relative mortality rate in women with T2DM versus women without T2DM is greater than in men with T2DM versus men without T2DM. This likely relates to a number of underlying determining factors. A systematic review of 35 prospective studies [8] found that in those with T2DM, all-cause and coronary heart disease mortality was greater in women by 17 and 97%, respectively. This effect has also been seen in type 1 diabetes, whereby mortality was 37% higher in women than in men [9]. In a recent meta-analysis, diabetes was associated with increased all-cause mortality; the relative risks were 1.59 in men and 2.00 in women [10], similar to our findings here.

Glycosylated haemoglobin (HbA1c), dyslipidemia, and hypertension were not found to be significantly predictive as these patients have been under active health management for a number of years and medication keeps these conditions for most patients within clinically significant control limits

Glucose-lowering therapies differ in their effects on cardiovascular mortality [11] and equality of access to these medications is essential to ensuring mitigation of sex differences in mortality. Further clarification of the factors associated with the sex difference in mortality is needed.

Multifactorial interventions at the patient, provider, healthcare system, and community level are required to reduce the mortality gap between men and women with diabetes. Awareness of this can be promoted at every interaction online, or face-to-face, that occurs between a woman with diabetes and their professional care team.

## Acknowledgements

### Conflicts of interest

There are no conflicts of interest.
